# A pilot open-label trial of minocycline in patients with autism and regressive features

**DOI:** 10.1186/1866-1955-5-9

**Published:** 2013-04-08

**Authors:** Carlos A Pardo, Ashura Buckley, Audrey Thurm, Li-Ching Lee, Arun Azhagiri, David M Neville, Susan E Swedo

**Affiliations:** 1 Department of Neurology, Pathology 627, Johns Hopkins University School of Medicine, 600 North Wolfe Street, Baltimore, MD, 21287, USA; 2 Pediatrics & Developmental Neuroscience Branch, National Institute of Mental Health, 10 Center Drive, Bldg 10/RM 1C250/MSC 1255, Bethesda, MD, 20892, USA; 3 Department of Epidemiology, Johns Hopkins University Bloomberg School of Public Health, 615 N. Wolfe Street, Room E6032, Baltimore, MD, 21205, USA

**Keywords:** Autism, Minocycline, Microglia, Neuroinflammation, Clinical trial, Cytokines, Chemokines, Metalloproteinases, Neurotrophins, BDNF

## Abstract

**Background:**

Minocycline is a tetracycline derivative that readily crosses the blood brain barrier and appears to have beneficial effects on neuroinflammation, microglial activation and neuroprotection in a variety of neurological disorders. Both microglial activation and neuroinflammation have been reported to be associated with autism. The study was designed to evaluate the effects of minocycline treatment on markers of neuroinflammation and autism symptomatology in children with autism and a history of developmental regression.

**Methods:**

Eleven children were enrolled in an open-label trial of six months of minocycline (1.4 mg/kg). Ten children completed the trial. Behavioral measures were collected and cerebrospinal fluid (CSF), serum and plasma were obtained before and at the end of minocycline treatment and were analyzed for markers of neuroinflammation.

**Results:**

Clinical improvements were negligible. The laboratory assays demonstrated significant changes in the expression profile of the truncated form of brain derived neurotrophic factor (BDNF) (*P* = 0.042) and hepatic growth factor (HGF) (*P* = 0.028) in CSF. In serum, the ratio of the truncated BDNF form and α-2 macroglobulin (α-2 M), was also significantly lower (*P* = 0.028) while the mature BDNF/α-2 M ratio revealed no difference following treatment. Only the chemokine CXCL8 (IL-8) was significantly different (*P* = 0.047) in serum while no significant changes were observed in CSF or serum in chemokines such as CCL2 (MCP-1) or cytokines such as TNF-α, CD40L, IL-6, IFN-γ and IL-1β when pre- and post-treatment levels of these proteins were compared. No significant pre- and post-treatment changes were seen in the profiles of plasma metalloproteinases, putative targets of the effects of minocycline.

**Conclusions:**

Changes in the pre- and post-treatment profiles of BDNF in CSF and blood, HGF in CSF and CXCL8 (IL-8) in serum, suggest that minocycline may have effects in the CNS by modulating the production of neurotrophic growth factors. However, in this small group of children, no clinical improvements were observed during or after the six months of minocycline administration.

**Trial registration:**

NCT00409747

## Background

Autism is a neurodevelopmental disorder that results in abnormalities of social communication and is associated with rigid and repetitive behaviors [1]. The spectrum of clinical features, including the onset and progression of neurobehavioral abnormalities, is quite heterogeneous. A subset of children with autism experiences a loss of language and social skills (so-called developmental regression). In this subset with regression, an accelerated disturbance of the neurobiological developmental trajectories may be taking place [2,3]. Previous neuropathologic and structural studies in patients with autism suggested the presence of ongoing innate neuroinflammatory mechanisms characterized by activation of microglia and astroglia together with abnormalities in cytokines and chemokines [4-7]. These findings highlighted the role of innate neuroimmune mechanisms and their potential to contribute to the establishment and progression of regressive features in autism by influencing processes of neuronal function, neuronal-glial interaction, and synaptic plasticity. Under normal conditions, microglia are integral components of brain homeostasis involved in synaptic plasticity, neurogenesis, axon guidance, and cortical organization [8-11]. However, in response to direct injury or to neuronal dysfunction, activated microglia can elaborate numerous cytokines and cytotoxins that can further damage neurons [12]. In autism, microglial activation has been shown to occur in regions of the central nervous system such as the cerebellum and the frontal and anterior cingular cortices [6,7], areas of the brain involved in the neurobehavioral abnormalities that characterize this disorder [13].

Minocycline is a semisynthetic derivative of tetracycline that readily crosses the blood brain barrier. In addition to being an antibacterial agent, it has direct neuroprotective effects [14] as well as anti-inflammatory properties. It also has a proven tolerability and safety profile for clinical use [15]. In recent years, it has become one of the most investigated therapeutic candidates for modulation of microglial activation and innate neuroinflammatory pathways in neurological disorders, although contradictory results have been obtained in recent clinical trials [16,17]. The exact mechanism by which minocycline modifies inflammation is not fully understood. The drug likely targets multiple inflammatory pathways via complex interactions with proteins integral to the inflammatory cascade [18]. This independent modulatory function on neuroinflammation is, at least in part, mediated via microglial inhibition [19].

The effect of minocycline on neuroinflammation and its potential role as a neuroprotectant has been evaluated in animal models of stroke, neurodegeneration, neuroimmunity, and neuroinfection [20]. Most of these studies showed that minocycline decreases microglial activation, modulates pathways involved in neuroinflammation, such as cytokine and chemokine networks (for example, interleukin-6 (IL-6), interleukin 1β (IL-1β) and tumor necrosis α (TNFα)), and reduces the activity of selected metalloproteinases (MMPs) [21,22]. The neuroprotective role of minocycline in these models has been attributed to anti-apoptotic and anti-oxidant properties [23]. Interestingly, minocycline appeared to regulate behavior and reduced the impact of stress on neuronal activation and working memory by modulation of microglial activation in a model of stress [24]. Based on the potential anti-inflammatory and neuroprotective properties shown by minocycline in animal models, therapeutic trials in humans have been carried out in a number of neuroinflammatory diseases such as multiple sclerosis [25] and neurodegenerative [26-28], and cerebrovascular diseases [29]. Recent studies in a mouse model of fragile X (Fmr1 KO), a neurodevelopmental disorder that shares some neurological and neurobiological features with autism, demonstrated that minocycline was able to promote dendritic spine maturation via inhibitory effects on MMP-9 expression and that these effects were accompanied by improvements in the behavioral performance of treated animals [30]. Furthermore, an open-label add-on treatment trial with minocycline in patients with fragile X showed behavioral benefits without major side effects [31]. On the other hand, while amyotrophic lateral sclerosis (ALS) studies in animal models showed great promise [32], as did the results of phase I and II clinical trials [16,17] a recently completed phase III randomized clinical trial showed adverse effects of the medication [17] in this disorder.

The potential usefulness of minocycline in a variety of neurological disorders involving neuroinflammation coupled with the above findings suggestive of innate neuroinflammatory pathways in autism, led to the hypothesis that minocycline could be useful in the treatment of patients that exhibit the regressive subtype of autism. Accordingly, we designed an open-label pilot trial study to evaluate the effects of minocycline on markers of neuroinflammation and to assess its impact on the clinical profile of children with regressive autism.

## Methods

### Study design and subjects

An open-label pilot trial of minocycline therapy for children with autism and a history of regression was approved by the National Institutes of Health (NIH) Institutional Review Board (http://NCT00409747). Consent for participating in the pilot trial was obtained from the parents or legal guardian. Minocycline dosage was calculated for each subject at 1.4 mg/kg per day. This dose was based on the Bonelli *et al*. 2004 study of minocycline in Huntington’s Disease, which used 100 mg/day in adult subjects [33]. Vitamin B6 at a dose of 0.6 mg/kg twice daily was given in conjunction with minocycline administration to mitigate the potential for vestibular side effects.

Subjects were referred and evaluated at the Intramural Autism Research Program of the National Institute of Mental Health, Bethesda, Maryland. Children were eligible for inclusion if they were between three and twelve years of age, met research criteria for a diagnosis of autism, had a history of developmental regression, and were stable on all behavioral and/or medical therapies. Autism diagnoses were made using the Autism Diagnostic Interview - Revised (ADI-R) [34] or a Toddler version, the Autism Diagnostic Observation Schedule [35], and clinical judgment based on DSM-IV criteria for autistic disorder. The diagnostic evaluations also included cognitive testing (described below), and information from the Vineland Adaptive Behavior Scales, Second Edition (VABS) [36]. Regression was defined as language loss (loss of at least three spontaneously meaningful words) and/or nonverbal communication/social loss (loss of more than one nonverbal communicative behavior). Regression histories were confirmed by the ADI-R and a modified Regression Validation Interview [37].

Subjects were excluded for known genetic defects, based on medical records or clinical genetics testing, prematurity of less than 32 weeks gestation or small for gestational age status, serious neurologic disorders (for example, cerebral palsy or uncontrolled epilepsy), evidence of renal insufficiency, hepatic disease or autoimmune disorder, or presence of a first degree relative with systemic lupus erythematous. Patients were also excluded if they were taking any medications that were contraindicated with either minocycline or vitamin B6.

Eleven children (9 boys, 2 girls; mean age 7.19 years; range 3 to 12 years) were enrolled in the trial. By clinical history, loss of social and communication skills had occurred at a mean age of 18.4 months (range 8 to 28 months). Ten children completed the six-month open-label trial; one child dropped out after three months because of parental concerns about side effects.

Clinical and behavioral assessments were performed at baseline and at post-treatment during the trial, and data are presented in Table 1. At baseline, eight participants were administered the Mullen Scales of Early Learning [38] and three participants were administered the Differential Ability Scales Second Edition (DAS-II) [39]. Since many of the children who were assessed with the Mullen Scales were out of age range or below the ‘floors’ of the standard scales, a nonverbal developmental quotient (NVDQ) was used to describe intellectual functioning. The NVDQ is the ratio of IQ of age equivalent/chronological age for the average of the nonverbal portions of the test. For the three participants administered the DAS-II, the nonverbal cognitive score was the standard score from the nonverbal reasoning domain.

**Table 1:** Patient sample description

**Subject**	**Pretreatment age (months)**	**Sex**	**Age at onset of regression (months)**	**Clinical assessment**				
				**Non-verbal IQ/DQ**	**CGI- baseline**	**CGI- post-treatment**	**Vineland- ABC baseline**	**Vineland-ABC post-treatment**
1	99	F	27	30	6	5	55	54
2^a^	64	M	13	60	5	4^a^	68	73^a^
3	69	M	12	33	5	5	45	44
4	153	M	19	77	5	5	56	55
5	91	M	8	58	3	3	60	56
6	66	M	18	103	4	4	70	71
7	128	F	15	25	5	5	55	46
8	112	M	14	33	5	5	53	56
9	49	M	18	59	4	4	66	63
10	38	M	22	90	4	3	70	66
11	83	M	18	32	5	5	48	57
				*M ± SD*	4.6 ± 0.8	4.4 ± 0.8	58.7 ± 8.8	58.3 ± 9.3
				*Effect Size*	-	−0.3	-	−0.1

^a^Denotes child that discontinued the study at 3 months.

Note: Effect size was calculated for the within-subjects effect using the following formula: ES = (M_Post_ - M_BL)_/SD_BL_). CGI, Clinical Global Impression.

The severity of core autism symptoms was rated at baseline and monthly intervals using the Clinical Global Impression Severity Scale (CGI-S) [40], and change was recorded with the CGI-Improvement (CGI-I). In addition, adaptive functioning was measured using the VABS. Parents were queried about medication side effects at monthly intervals.

Serum, plasma and cerebrospinal fluid (CSF) samples were obtained at baseline and following six months of minocycline administration. CSF was obtained by lumbar puncture under fasting conditions, while the child was sedated with propofol. Venous blood samples were obtained at the same time as the CSF and aliquots of both were sent for immediate analysis of routine laboratory tests at NIH or stored (−80°C) for research analyses. Subjects also underwent monthly blood tests for evaluating potential hematological, metabolic, or hepatic side effects.

The cytokines TNFα, IFN-γ, IL-1α, IL-1β, IL-2, IL-3, IL-4, IL-5, IL-6, IL-7, IL-10, IL-12(p40), IL 12(p70), IL-13, IL-15, GM-CSF and the chemokines CCL2 (MCP-1), CCL3 (MIP-1α), CCL5 (Rantes), CXCL8 (IL-8), CCL11 (Eotaxin) and CXCL10 (IP-10) were measured in the serum and CSF at baseline and after six months treatment with minocycline. Additional assays included comparisons of baseline and six months concentrations of plasma and CSF leptin, CD40 ligand and neurotrophic factors such as brain neurotrophic growth factor (BDNF), hepatic growth factor [HGF], and glial derived growth factor [GDNF]. MMPs (MMP-1, MMP-3, MMP-7, MMP-8 and MMP-9) were also measured in plasma.

All analytes were measured by multiplexed bead array assays (Luminex™) techniques with exception of the pro-form (pro-BDNF), truncated form (truncated-BDNF) and mature form of BDNF (mature-BDNF) that were measured by immunoblot analysis of serum samples using an antibody that recognized all three isoforms (Santacruz Biotechnology, Inc., Dallas, Texas, USA, cat# SC546). The relative presence of the pro-BDNF, truncated-BDNF and mature-BDNF in serum was determined by densitometric analysis and its presence was established as a ratio in which α-2 macroglobulin was used for normalization. Multiplexed assay kits and beads were obtained from commercial sources (for example, BioRad™, Invitrogen™, R&D systems™ and Millipore™) and procedures followed manufacturers’ recommendations. Blinded samples were measured in duplicates and blank values subtracted from all readings. Measurements and data analysis of all assays were performed with the Luminex-200™ system in combination with Luminex manager software (Bioplex Manager 5.0, Bio-Rad, Hercules, CA, USA). Our biomarker quality assurance program used standard operating procedures to review results for unexpected or unacceptable variance (evidence of bead clumping, coefficients of variation greater than 20%, unusual distributions of values, outliers more than 4 SD from the mean).

### Statistics

The Mann–Whitney *U*-test was used to compare pre-/post-treatment differences in analyte concentrations in serum, plasma and CSF. Statistical significance level was set at 0.05.

## Results

### Clinical outcomes

Clinical improvements were negligible, with CGI-S scores remaining stable and only two of ten children demonstrating ‘minimal improvement’ on the CGI-I. The VABS composite scores also showed little or no change (Table 1). Adverse events reported by parents (Table 2) included gastrointestinal and upper respiratory (‘colds’) symptoms (Table 2). Side effects were deemed problematic for three subjects. One child (subject 7) was reported to have weight gain, appetite increase, diaper rash, pica, and concerns about her teeth becoming discolored (could not differentiate from developmental defects of enamel). Another child (subject 8) was observed to have persistent, benign hematuria. The third subject (subject 2), a minimally verbal child, was reported to have had several episodes of crying and holding his head, which the mother reported to be consistent with headache. This child was withdrawn from the study at three months because of parental concerns about the adverse events.

**Table 2:** Reported health problems during minocycline treatment

***Subject***	***URI***^***a***^	***GI***^***b***^	***Others***
1	X		Hematuria, weight gain, hyperactivity, urinary tract infection
2^c^	X		Increased aggression and head-banging, c/o head hurting
3		X	Otitis media
4	X		Epistaxis, increased sensitivity to lights
5	X	X	Increased aggression
6	X		
7			Teeth staining; increased appetite, aggression and ritualistic behavior, weight gain, PICA
8	X	X	Microscopic hematuria
9		X	
10	X	X	
11	X	X	

^a^URI, Upper respiratory infection symptoms.

^b^GI, Gastrointestinal symptoms included nausea, vomiting, and/or diarrhea on at least one occasion.

^c^Subject withdrew at three months.

### Cytokines and Chemokine profiles

**Figure 1:**
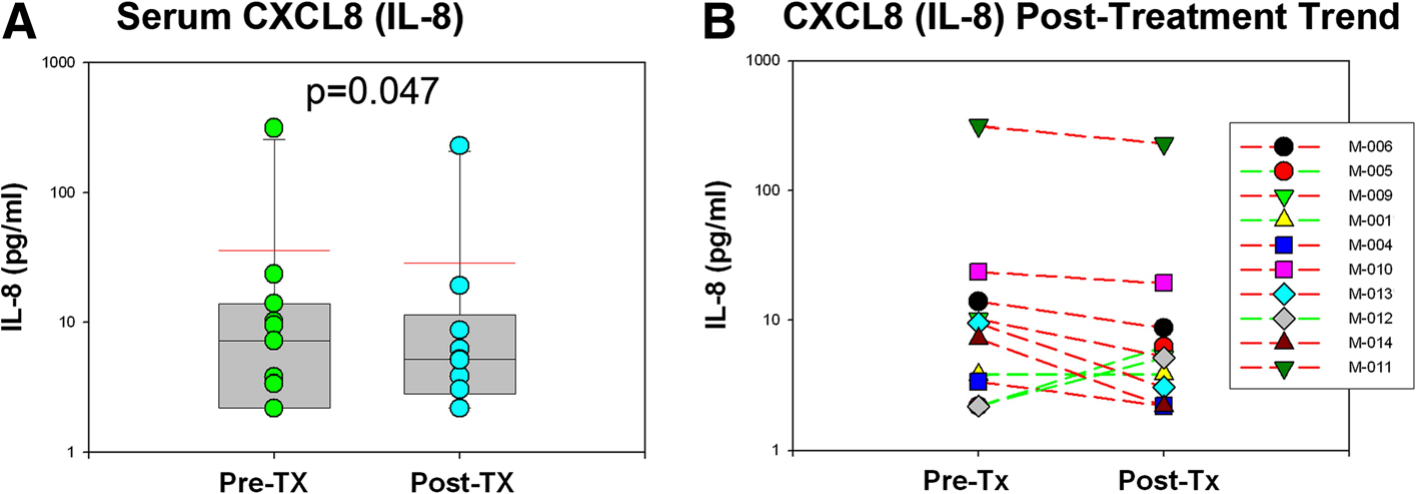
**CXCL8 (IL-8) Post-treatment trend in serum. **(**A**) Serum levels of CXCL8 (IL-8) were significantly lower after treatment with minocycline. (*P* = 0.047). (**B**) The trend profile of CXCL8 (IL-8) in serum showed a lowering effect of treatment in seven of ten patients (red lines) while only three of ten (green lines) showed an increase.

There were no significant changes in the quantitative assessment of cytokines and chemokines in both serum and CSF as measured by multiplexed microbead array technology (Luminex™). Expression of chemokines, such as CCL2 (MCP-1) or cytokines such as TNF-α, CD40L, IL-6, IFN-γ and IL-1β, well recognized immune mediators implicated in inflammatory mechanisms, were not affected in serum or CSF when pre- and post-treatment levels of these proteins were compared (Table 3). Only the chemokine CXCL8 (IL-8) in serum was significantly reduced after treatment (*P* = 0.047) (Figure 1A); seven of ten patients had reduction in the serum level of CXCL8 (IL-8) (Figure 1B).

**Table 3:** **Treatment effect on cytokines, chemokines, metalloproteinases and growth factors**^**a**^

**Analyte**	**Cerebrospinal fluid (CSF)**		**Serum**		**Plasma**	
	**Z**	***P***	**Z**	***P***	**Z**	***P***
TNFα	−0.45	0.66	−1.78	0.074		
IL-6	−0.36	0.72	−1.75	0.08		
CCL2 (MCP-1)	−1.78	0.075	−1.27	0.24		
CCL3 (MIP-1α)	−0.18	0.86	−0.76	0.45		
CCL5 (RANTES)	−0.73	0.47	0.66	0.61		
CXCL8 (IL-8)	−0.45	0.66	−1.99	**0.047**		
BDNF^b^	−2.03	**0.042**			−0.76	0.45
Truncated-BDNF/α-2 M^c^			−2.19	**0.028**		
Mature-BDNF/α-2 M ^c^			−0.87	0.386		
CD40L	−0.89	0.37			−1.33	0.18
GDNF	−0.37	0.71			−1.83	0.07
HGF	−2.19	**0.028**			−1.25	0.21
Leptin	−1.48	0.14			−0.27	0.79
MMP-1					−1.07	0.29
MMP-3					−0.15	0.88
MMP-7					−1.89	0.059
MMP-8					−0.05	0.96
MMP-9					−1.38	0.17

^a^Selected analytes, Mann–Whitney *U*-test.

^b^Quantified by Luminex technique. In CSF only the truncated-BDNF form appears to be detectable in immunobotting experiments.

^c^Quantified by immunoblotting and densitometry analysis. Significance was calculated based on ratio BDNF isoform/α-2 M.

### Growth factor profiles

**Figure 2:**
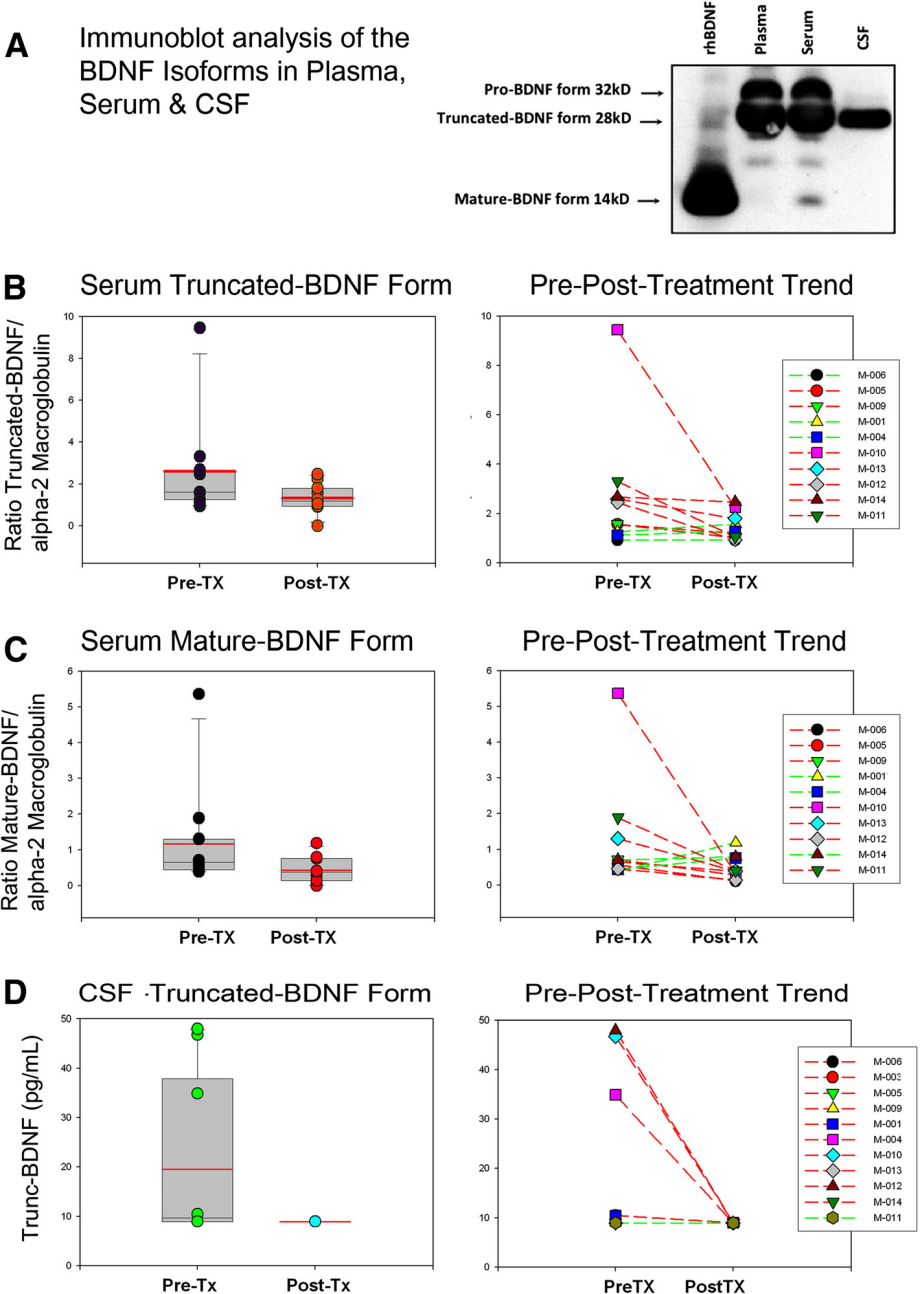
**Profiles of expression of brain derived neurotrophic factor in serum and cerebrospinal fluid. **(**A**) Immunoblot analysis of brain derived neurotrophic factor (BDNF) isoforms in serum disclosed the presence of the pro-form (32 kD), truncated-form (28 kD) and mature form (14 kD) while plasma disclosed mostly the presence of the BDNF pro-form and truncated-form. The truncated-BDNF form was the only isoform seen in cerebrospinal fluid (CSF). (**B**)The BDNF truncated-form was significantly decreased after treatment with minocycline (*P* = 0.028) a trend that was observed in six of ten patients (red lines). (**C**) Although the mature form appeared to be decreased after treatment the difference pre- and post-treatment did not reach significance (*P* = 0.38). (**D**) BDNF in CSF measured by multiplexed array technique, which detects mostly the truncated-BNDF isoform, was significantly lowered after treatment (*P* = 0.042).

**Figure 3:**
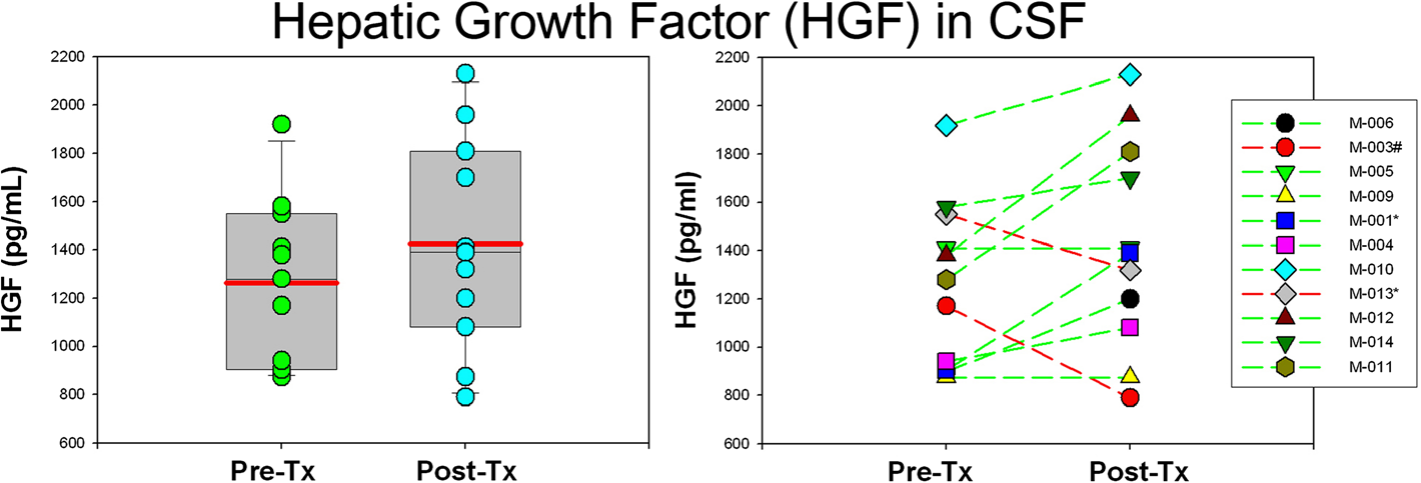
**The levels of hepatic growth factor (HGF) in cerebrospinal fluid (CSF) were significantly increased following treatment with minocycline.** (*P* = 0.028).

Profiling of BDNF used Luminex™ and immunoblotting techniques. Immunoblot analysis, which facilitates the assessment of the BDNF isoforms, showed that while the pro-BDNF, truncated-BDNF and mature-BDNF isoforms are identified in serum, plasma samples are devoid of the mature-BDNF form, an observation that reflects the release of the mature-BDNF form from platelets during clot formation. Interestingly, similar immunoblot studies showed that only the truncated-BDNF isoform is detected in the CSF (Figure 2A), a finding that demonstrates that only such form is measurable by the available Luminex or ELISA techniques. Immunoblot analysis of the BDNF isoforms in serum showed the truncated-BDNF/α-2 M ratio, a measure of the relative presence of such isoform was significantly lower following minocycline treatment at six months (*P* = 0.028) (Figure 2B) while the mature-BDNF/α-2 M ratio demonstrated no difference (*P* = 0.38) (Figure 2C). Interestingly, BDNF measured by Luminex™ technique in CSF, (assumed to be the truncated-BDNF form), showed a significantly lower concentration post-treatment (*P* = 0.042) (Figure 2D). The plasma BDNF concentration measured by Luminex™ technique, which reflects the presence of pro- and truncated-BDNF isoforms combined, did not show difference after treatment (*P* = 0.45). Contrary to BDNF, the growth factor HGF, (and ligand for MET), was found to be significantly increased in the CSF after treatment (*P* = 0.028) (Figure 3) but was not significantly different in serum. The CSF concentration of GDNF showed no difference after minocycline treatment.

### Metalloproteinases

**Figure 4:**
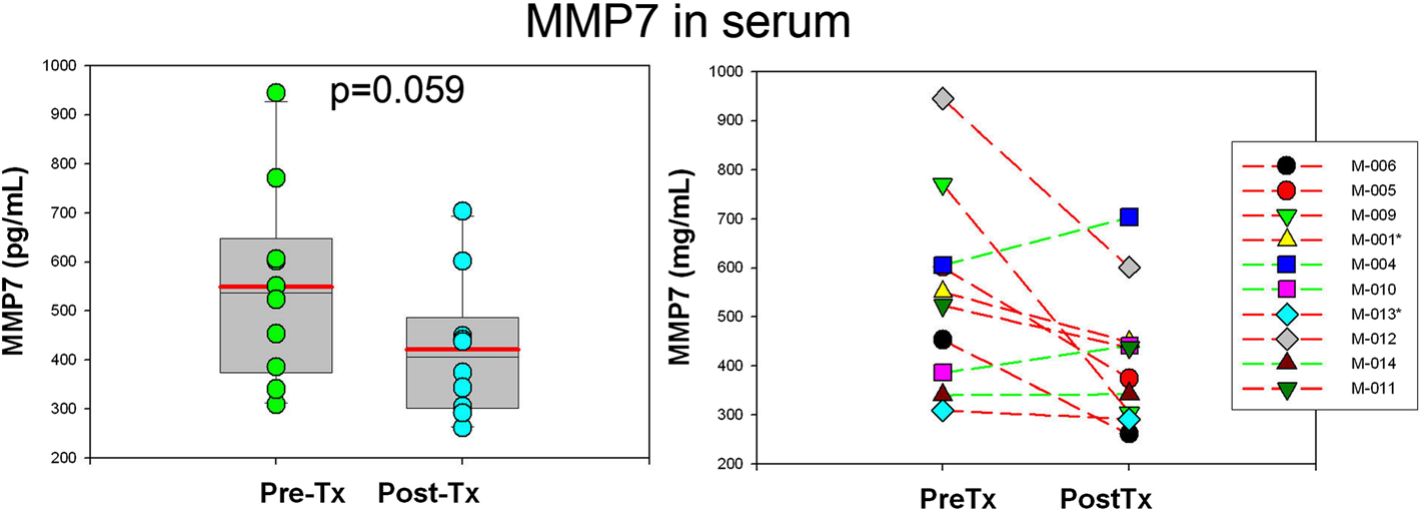
**A post-treatment lowering effect on MMP-7 was observed in seven of ten patients (red lines), but despite this trend it did not reach statistical significance.** (*P* = 0.059).

No significant post-treatment changes were seen in the profiles of MMPs (MMP-1, MMP-3, MMP-7, MMP-8 and MMP-9) in either CSF or plasma, although there was a trend towards change in plasma MMP7 (*P* = 0.059) (Figure 4), one of the MMPs that appears to be a target for minocycline.

## Discussion

The trial revealed no significant effects of minocycline on the profile of immune mediators implicated in inflammatory mechanisms in either the serum or CSF of treated children with the exception of a reduction in the levels of CXCL8 (IL-8), a chemokine that appears to have neuroprotective effects [41] and regulates the release of MMPs [42]. A hypothesized reduction of immune modulators associated with monocyte trafficking or microglia function such as CCL2 (MCP-1) or certain MMPs, was not proven by this clinical trial. Concentrations of MMPs in this study were unaffected after treatment with minocycline and only MMP-7 disclosed a decreasing trend (*P* = 0.059) while MMP-9, a putative target of minocycline effect [43], was unchanged after treatment.

Interestingly, changes in the profiles of some growth factors suggest that minocycline exerted biological effects that were not translated into behavioral or neurological changes. The changes observed in the pre-/post-treatment profiles of truncated-BDNF isoforms in CSF and blood and HGF in CSF, are intriguing as they may suggest that higher doses of minocycline could potentially modulate the proteolytic processing of some neurotrophic growth factors. BDNF pro-form and truncated-form appear to be critical for mechanisms of synaptic plasticity. Both forms are not necessarily inactive intermediates in the biosynthesis of BDNF but rather biologically active forms with opposite functions with respect to the mature-BDNF form that may be involved in the neurobiological abnormalities that underlie cognitive dysfunction [44-47]. Interestingly, an altered balance of the processing of BDNF in autism was demonstrated recently by studies of the fusiform gyrus of the brain of patients with autism that disclosed abnormal processing of the pro- and truncated-BDNF forms [48]. HGF acts as the ligand for cMET, a receptor protein kinase in which the functional promoter variant of the gene has been implicated as a risk for autism and its social and communication phenotypes [49,50].

Overall, there were no significant improvements in the clinical and behavioral measurements of this small group of children with autism and regressive features during the six-month treatment period. Two of the younger children appeared to have minimal improvements on the CGI. However, objective measures of nonverbal cognitive ability, adaptive behavior and overall autism symptoms showed no significant changes during open-label administration of minocycline.

The lack of a meaningful clinical effect requires us to reject the hypothesis that a daily dose of 1.4 mg/kg of minocycline will produce improvements in the neurobiological trajectories of individuals with autism. The lack of clinical and biological effects in our patient population may reflect: 1) limitations of the study design, including wide age range; 2) the complexity of neurobiological mechanisms involved in autism pathogenesis; 3) the relatively low dose of the medication used in this trial as compared with clinical trials conducted with young adults or elderly patients; or 4) differences in drug metabolism and neurobiological effects of the medication in young children. While a higher dose might prove useful in changing the CSF markers of inflammation, it would also be associated with an increased risk of toxicity. The prominence of adverse effects in this small sample of young children with autism suggests that more aggressive treatment regimens would not be tolerable and might be anticipated to have an unfavorable risk: benefit ratio.

## Conclusions

No clinical improvement was observed after minocycline treatment in this small pilot open-trial study of children with autism and regressive features. Interestingly, changes in the pre- and post-treatment profiles of some markers, including BDNF isoforms in CSF and blood, HGF in CSF, and CXCL8 (IL-8) in serum, suggest that minocycline might have effects in the CNS by modulating the production of neurotrophic growth factors.

## Abbreviations

ALS: Amyotrophic lateral sclerosis; BDNF: Brain derived neurotrophic factor; CGI-I: Clinical Global Impression Improvement Scale; CGI-S: Clinical Global Impression Severity Scale; CSF: Cerebrospinal fluid; GDNF: Glial derived growth factor; HGF: Hepatic growth factor; MMPs: Metalloproteinases; NIH: National Institutes of Health; NVDQ: Nonverbal developmental quotient; VABS: Vineland Adaptive Behavior Scales; α-2 M: α-2 macroglobulin.

## Competing interests

The authors declare that they have no competing interests.

## Authors’ contributions

CAP, SS, AT and DN conceived and participated in the design of the study. AB and AT participated in the evaluation and clinical care of patients. AA carried out immunoassays and quantitative immunoblots. LCL performed the statistical analysis. CAP, AB, AT and SS drafted the manuscript. The manuscript was reviewed and approved by all authors.
